# Fluctuations of Spleen Cytokine and Blood Lactate, Importance of Cellular Immunity in Host Defense Against Blood Stage Malaria *Plasmodium yoelii*

**DOI:** 10.3389/fimmu.2019.02207

**Published:** 2019-09-25

**Authors:** Takashi Imai, Kazutomo Suzue, Ha Ngo-Thanh, Suguri Ono, Wakako Orita, Haruka Suzuki, Chikako Shimokawa, Alex Olia, Seiji Obi, Tomoyo Taniguchi, Hidekazu Ishida, Luc Van Kaer, Shigeo Murata, Keiji Tanaka, Hajime Hisaeda

**Affiliations:** ^1^Department of Infectious Diseases and Host Defense, Gunma University Graduate School of Medicine, Maebashi, Japan; ^2^Department of Parasitology, Graduate School of Medical Sciences, Kyushu University, Fukuoka, Japan; ^3^Department of Parasitology, National Institute of Infectious Diseases, Tokyo, Japan; ^4^Center for Medical Education, Graduate School of Medicine, Gunma University, Maebashi, Japan; ^5^Department of Pathology, Microbiology and Immunology, Vanderbilt University Medical Center, Nashville, TN, United States; ^6^Laboratory of Protein Metabolism, Graduate School of Pharmaceutical Sciences, The University of Tokyo, Tokyo, Japan; ^7^Laboratory of Protein Metabolism, Tokyo Metropolitan Institute of Medical Science, Tokyo, Japan

**Keywords:** malaria, T cell, CD8 T cell, CD4 T cell, macrophage, erythroblast

## Abstract

Our previous studies of protective immunity and pathology against blood stage malaria parasites have shown that not only CD4^+^ T cells, but also CD8^+^ T cells and macrophages, are important for host defense against blood stage malaria infection. Furthermore, we found that *Plasmodium yoelii* 17XNL (PyNL) parasitizes erythroblasts, the red blood cell (RBC) precursor cells, which then express MHC class I molecules. In the present study, we analyzed spleen cytokine production. In CD8^+^ T cell-depleted mice, IL-10 production in early stage infection was increased over two-fold relative to infected control animals and IL-10^+^ CD3^−^ cells were increased, whereas IFN-γ production in the late stage of infection was decreased. At day 16 after PyNL infection, CD8^+^ T cells produced more IFN-γ than CD4^+^ T cells. We evaluated the involvement of the immunoproteasome in induction of immune CD8^+^ T cells, and the role of Fas in protection against PyNL both of which are downstream of IFN-γ. In cell transfer experiments, at least the single molecules LMP7, LMP2, and PA28 are not essential for CD8^+^ T cell induction. The Fas mutant LPR mouse was weaker in resistance to PyNL infection than WT mice, and 20% of the animals died. LPR-derived parasitized erythroid cells exhibited less externalization of phosphatidylserine (PS), and phagocytosis by macrophages was impaired. Furthermore, we tried to identify the cause of death in malaria infection. Blood lactate concentration was increased in the CD8^+^ T cell-depleted PyNL-infected group at day 19 (around peak parasitemia) to similar levels as day 7 after infection with a lethal strain of Py. When we injected mice with lactate at day 4 and 6 of PyNL infection, all mice died at day 8 despite demonstrating low parasitemia, suggesting that hyperlactatemia is one of the causes of death in CD8^+^ T cell-depleted PyNL-infected mice. We conclude that CD8^+^ T cells might control cytokine production to some extent and regulate hyperparasitemia and hyperlactatemia in protection against blood stage malaria parasites.

## Introduction

Malaria is one of the most deadly infectious diseases, with 219 million cases and 435,000 deaths per year worldwide^1^ The contribution of CD8^+^ T cells to host defense against blood stage malaria infection has long remained controversial. Many studies have reported a protective role for CD8^+^ T cells against liver stage malaria infection, so this protective role for CD8^+^ T cells appears to be established (1–5). However, the role of CD8^+^ T cells in blood stage malaria infection remains controversial. This controversy is further compounded by the use of different rodent parasites with differential effects. For example, infection of C57BL/6 mice with *Plasmodium yoelii* 17XL (PyL) causes lethal infection through high parasitemia (6). Contrastingly, *P. yoelii* 17XNL (PyNL) infection results in a self-limiting infection (7). However, *P. berghei* ANKA (PbA) infected-mice develop lethal cerebral malaria, despite low parasitemia (8). The combination of different mouse strains and parasites results in different outcomes in the course of infection. Each combination demonstrates a different potential contribution of CD8^+^ T cells to host defense or immunopathology.

For example, we and others confirmed that CD8^+^ T cells are involved in development of experimental cerebral malaria (3, 9, 10). CD8^+^ T cells have two primary functions after antigen presentation and activation by dendritic cells (DCs). First, CD8^+^ T cells activate macrophages by producing IFN-γ, and secondly confer antigen-specific cytotoxicity against MHC class I molecule-expressing cells (11). Parasite antigens are cross-presented by brain endothelial cells to CD8^+^ T cells in the experimental cerebral malaria model, in which C57BL/6 mice are infected with PbA (12). CD8^+^ T cells attack parasite antigen-presenting vascular endothelial cells, leading to disruption of the blood brain barrier and subsequent development of experimental cerebral malaria (13). This is a mechanism by which CD8^+^ T cells contribute to immunopathology in experimental cerebral malaria.

Contrastingly, in rodent malaria such as *P. chabaudi*, the PyNL and PyL live vaccination defense model demonstrated that CD8^+^ T cells may contribute to blood stage parasite elimination (7, 14–18). Conversely, some studies suggested that CD8^+^ T cells may not contribute to protection against blood stage malaria parasites (19, 20). Red blood cells (RBCs), which are infected by blood stage malaria parasites, do not express MHC class I molecules as do normal cells such as hepatocytes, prohibiting recognition by CD8^+^ T cells. This phenotype has been considered to be the reason that CD8^+^ T cells do not contribute to protection against blood stage malaria, but our present finding that erythroblasts infected with PyNL express MHC class I, indicates the possibility that CD8^+^ T cells interact with parasitized erythroblasts and contribute to protection against blood stage malaria infection (7, 21).

We demonstrated in the live vaccine model that CD8^+^ T cells or CD4^+^ T cells from mice infected with PyNL boosted twice with PyL (immune CD8^+^ or CD4^+^ T cells) confer protection against PyL infection in recipient mice inoculated with CD8^+^ or CD4^+^ T cells from infected mice (16). We analyzed the characteristics of immune T cells. When immune T cells from *IFN-*γ^−/−^ donor mice were used, no protection against PyL was observed in either immune *IFN-*γ^−/−^ CD8^+^ T cells or immune *IFN-*γ^−/−^ CD4^+^ T cells, suggesting that IFN-γ is essential in protection against PyL. Indeed, many studies showed that IFN-γ plays a central role in immunity, inducing MHC class I molecules, Fas/FasL and induction of immunoproteasome related factors (22). However, the contribution of these downstream factors to host defense against blood stage infection remains incompletely understood. Contrastingly, when immune T cells from cytotoxic molecule Perforin (PFN) KO mice or FasL mutant (GLD) mice were used as donor cells, CD4^+^ T cells successfully conferred complete protection against PyL. However, protection by CD8^+^ T cells was not sufficient, and some mice died. These findings indicated that protection against PyL by immune CD4^+^ T cells does not require cytotoxic molecules, but that CD8^+^ T cells partially require cytotoxic molecules in the live vaccine model (7, 16). An additional study demonstrated that mice depleted of CD8^+^ T cells were unable to develop a defense against PyNL, resulting in 30–80% mortality. These findings raise a paradox, in which CD4^+^ T cells and NK cells are still present when CD8^+^ T cells are depleted, raising the possibility that the former cells might be a relevant source of IFN-γ production in the face of CD8^+^ T cell depletion.

IFN-γ is produced by other cells in addition to CD8^+^ T cells and CD4^+^ T cells. However, the role of other cytokines remains incompletely understood in blood stage malaria. It is currently unclear whether CD8^+^ T cells act as killer T cells, and do not contribute to cytokine production, whereas CD4^+^ T cells act as helper T cells, and contribute to immune regulation mainly through cytokine production in the blood stage malaria infection.

IFN-γ is known to convert the constitutive proteasome to the immunoproteasome (22). Prior studies have demonstrated that antigen processing by the immunoproteasome is better than that of the constitutive proteasome (23). It is unclear whether CD8^+^ T cells induced in the live vaccine model are immunoproteasome dependent. Additionally, we reported an interaction between FasL on CD8^+^ T cells and Fas on target cells, although it is unclear if the same phenomenon occurs when using the Fas mutant LPR mouse (24). In addition to Tim-4, MFG-E8 (25) recognizes PS, and the role of this molecule in host defense against Py infection is also unknown. Furthermore, it is unclear whether the hyperparasitemia observed in CD8^+^ T cell-depleted PyNL infection is the only cause of death or an additional factor contributing to lethality. We conducted the present study to address these gaps in knowledge.

## Materials and Methods

### Mice

Male and female C57BL/6 (B6) mice and C57BL/6JSlc-LPR (Fas^lpr^) mice were obtained from SLC (Hamamatsu, Japan) or Kyudo (Tosu, Japan). There were no significant differences between male and female mice in our experiments. Proteasome activator PA28 KO (*PA28*α^−/−^*/*β^−/−^) and immunoproteasome subunit LMP2 KO (*LMP2*^−^^/−^) mice were established by our group (26, 27). Immunoproteasome subunit LMP7 KO (*LMP7*^−/−^) mice (28) on the B6 background were kindly provided by Dr. Fehling (University Clinics Ulm). Milk fat globule-EGF factor 8 protein (MFG-E8) KO (*MFG-E8*^−^^/−^) mice (25) were kindly provided by Dr. Hanayama (Kanazawa Univ.). All mice were maintained under specific-pathogen-free conditions. Experiments were performed in mice aged 8–12 weeks.

### Rodent Malaria Parasite *Plasmodium yoelii* Infection

The clonal line of blood-stage *P. yoelii* 17XNL (PyNL) and 17XL (PyL) parasites originating from Middlesex Hospital Medical School, University of London, 1984, were generous gifts from Dr. M. Torii (Ehime University), and the generation of PyNL–GFP has been described previously (21). Blood-stage parasites were obtained after fresh passage of frozen stock through a donor mouse, 4–5 days after inoculation. Mice were infected intraperitoneally with parasitized red blood cells (pRBCs), with 25,000 pBRCs for PyNL and 50,000 pRBCs for PyL.

### Antibodies and Reagents

All antibodies were purchased from BD Pharmingen (Franklin Lakes, NJ, USA), eBioscience (San Diego, CA, USA), or BioLegend (San Diego, CA, USA). AF488-conjugated anti-CD4 (clone: GK1.5), PE-Cy7-conjugated anti-CD8a (clone: 53-6.7), PE-conjugated anti-CD3 (clone: 17A2), PE-, PE-Cy7-, and APC-conjugated anti-TER119 (clone: Ter119), PE- and PE-Cy7-conjugated anti-CD11b (clone: M1/70), APC-conjugated anti-IFN-γ (clone: XMG1.2), APC-Cy7-conjugated anti-IL-10 (clone: JES5-16E3) purified anti-CD16/32 (clone: 2.4G2) antibodies were used for blocking. Propidium iodide (Sigma, St. Louis, MO, USA) or 7-amino-actinomycin D (BioLegend) were used for dead cell staining when dead cells were excluded from analysis. Annexin V (BD Pharmingen) was used to stain phosphatidylserine (PS). A CD8α^+^ T cell isolation kit, CD11c, or CD11b Micro beads (Miltenyi Biotech, Bergisch Gladbach, Germany) were used for MACS cell purification (Miltenyi Biotech). A PKH26 Red Fluorescent Cell Linker Kit for General Cell Membrane Labeling was purchased from Sigma-Aldrich. The culture medium was RPMI 1640 (Sigma) containing 10% fetal bovine serum, 2 mM L-glutamine, 1 mM sodium pyruvate, 0.1 mM nonessential amino acids, penicillin–streptomycin, and 2-mercaptoethanol. Mouse ELISA kits for IFN-γ, IL-2, IL-3, IL-10, IL-17A, TNF-α, GM-CSF, were purchased from BioLegend. The mouse ELISA kit for MFG-E8 was purchased from R&D. ELISAs were conducted according to the manufacturer's protocol.

### Flow Cytometry

Cell suspensions from peripheral blood and spleen without RBC lysis were used in order to analyze erythroid cells (Ter119^+^) only for **Figures 7C,D**, for the other analysis for WBC, RBC was lysed with ACK lysing buffer. Samples were incubated with anti-CD16/32 antibody (Fc block) and stained with fluorochrome-labeled antibodies. Isotype control antibodies were also used to evaluate specific staining. Cells were analyzed with a FACSCalibur, FACSVerse, or FACSAria II flow cytometer (Becton Dickinson, San Jose, CA, USA), and data were analyzed with FlowJo software (Treestar, Ashland, OR, USA).

### Measurement of Blood Lactate and Lactate Administration

Blood lactate was measured in tail vein blood by using Lactate Pro2 (arkray, Kyoto, Japan). Lactate [L-(+)-Lactic acid, Sigma] was dissolved in RPMI1640 to a concentration of 10 mg/ml and 5 or 10 mg per mouse was administered intraperitoneally (i.p.) followed by monitoring of the blood lactate concentration.

### Cell Separation

CD8^+^ T cells: RBCs were removed from the spleen with ACK lysing buffer. Cells were Fc-blocked, then negatively sorted twice with a column using a CD8α^+^ T cell isolation kit [CD4, CD11b, CD11c, CD19, CD45R (B220), CD49b (DX5), CD105, anti-MHC-class II, TER119, TCRγ/δ], followed by positive sorting with anti-CD8 microbeads. The purity of the separated CD8^+^ T cells was approximately 95%. RBCs: Peripheral blood samples were added to a CF11 cellulose column (Whatman, Kent, UK) for depletion of WBCs, and allowed to flow through under gravity. pRBCs were then separated using Percoll density gradient centrifugation (GE Healthcare Bio-Sciences, Piscataway, NJ, USA).

### *In vivo* Depletion of T Cell Subsets, Prime–Boost Live Vaccination, and Cell Transfer Experiments

**Figure 1:**
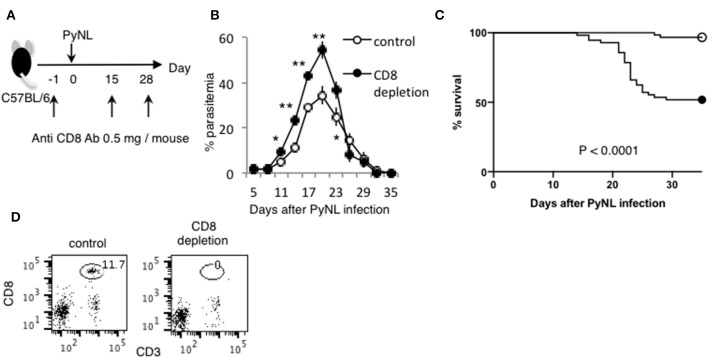
CD8^+^ T cells contribute to protection against PyNL infection. **(A)** C57BL/6 mice were injected with anti CD8 Ab or control Ab (0.5 mg/mouse) 1 day before and 15, 28 days after PyNL infection. **(B)** Parasitemia and **(C)** survival rate after PyNL infection. Data were pooled from 11 independent experiments [control group: *N* = 60, white circle; anti-CD8 (CD8^+^ T cell depletion): *N* = 56, black circle]. *P* < 0.0001. Parasitemia values are expressed average ±SD. **(D)** Depletion of CD8^+^ T (CD8^+^ CD3^+^) cells was confirmed by FACS. *P < 0.05, **P < 0.01.

Depletion of CD4^+^ or CD8^+^ T cells was performed as previously described (29). Briefly, mice were intraperitoneally administered 0.5 mg anti-CD4 (clone: GK1.5), anti- CD8α (clone: 2.43) antibodies 1 day before and 15 and 28 days after PyNL infection (Figure 1A). The depletion of each T-cell subset was verified by flow cytometry, and >99% of the appropriate cell subset was depleted in the peripheral blood 24 h after inoculation (Figure 1D) (7). The protocols for the prime–boost live vaccination and cell transfer are shown in **Figure 6C**. CD8^+^ T cells were isolated from WT and immunoproteasome related-molecule KO (*LMP2*^−/−^*, LMP7*^−/−^*, PA28*^−/−^) mice infected with PyNL (25,000 pRBCs) after two boosts with PyL (50,000 pRBCs) at 6 and 9 weeks after primary PyNL infection. Subsequently, 1 × 10^7^ purified cells were transferred to recipient X-ray-irradiated (5.5 Gy) WT mice. Recipient mice were infected with PyL (50,000 pRBCs) 1 week after cell transfer.

### *In vitro* Phagocytosis Assay

Collected pRBCs were washed twice with medium. Cells (2 × 10^7^ cells/ml) were stained with 250 nM carboxyfluorescein succinimidyl ester (CFSE: Molecular Probes; Life Technologies, Carlsbad, CA, USA) for 1 min. Staining was halted by addition of fetal calf serum, and cells were washed three times with medium. Splenic CD11b^+^ macrophages from uninfected mice were sorted with the MACS cell separation system, and subsequently labeled with PKH26 according to the manufacturer's instructions. Splenic CD11b^+^ macrophages (1 × 10^5^ cells) were co-cultured with CFSE-labeled pRBCs or normal RBCs in a 1:20 ratio, at a final volume of 200 μl for 4 h at 37°C in a CO_2_ incubator with culture medium. Following co-culture, non-ingested RBCs were removed with ACK lysing buffer. The capacity of macrophages to phagocytize CFSE-labeled pRBCs or normal RBCs was analyzed with a FACSCalibur flow cytometer.

### Statistical Analysis

Two sets of data (control vs. experimental group) were compared, and a Mann–Whitney *U*-test was used for statistical analysis. A *p* < 0.05 was considered to be statistically significant. Significant differences in survival were tested with a log-rank test using Kaplan–Meier survival curves.

## Results

### IFN-γ Production by CD8^+^ T Cells or CD4^+^ T Cells

**Figure 2:**
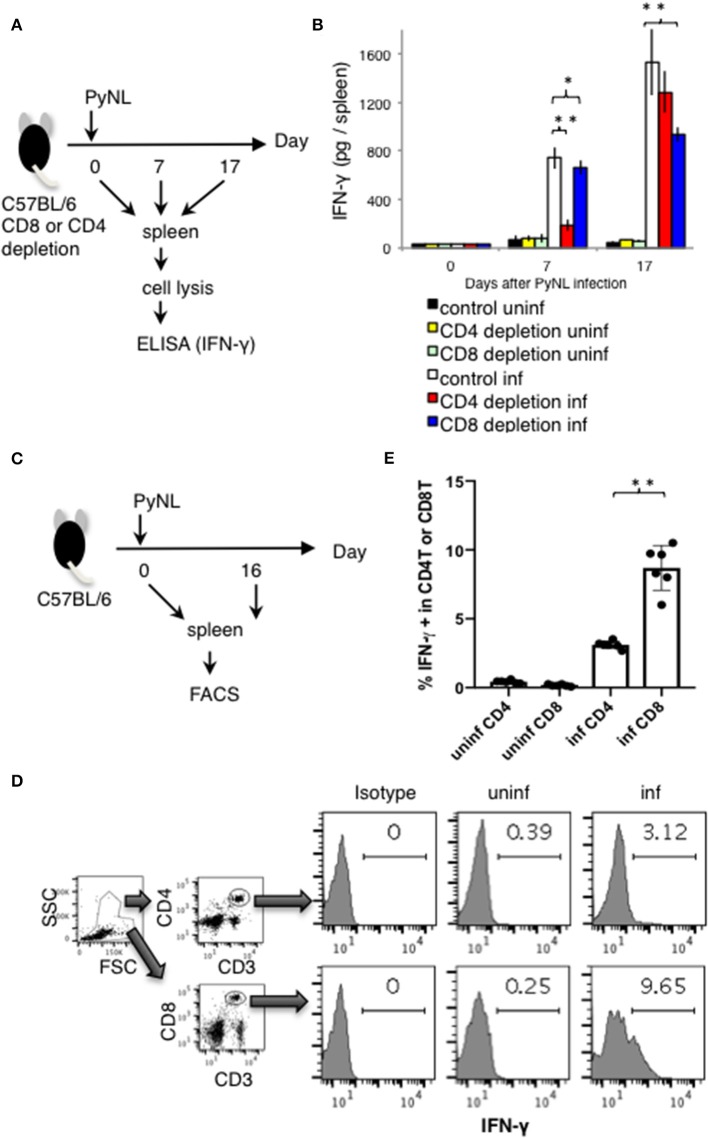
IFN-γ production from CD4^+^ or CD8^+^ T cells from PyNL infected mice and from CD4^+^ or CD8^+^ T cell-depleted mice *ex vivo*. **(A)** CD4^+^ or CD8^+^ T cells were depleted with antibody administration at 1 day before and 15 days after PyNL infection. Spleens were collected from day 0, 7, and 17 after infection. Cells were lysed and centrifuged to remove cell debris, and supernatants were subjected to ELISA (IFN-γ). **(B)** One representative result from four independent experiments is shown, and is expressed as average ± SD. *N* = 3–6. **(C)** C57BL/6 mice were infected with PyNL at day 0. Spleens were collected at day 0 and day 16 after PyNL infection. CD4^+^ or CD8^+^ T cells were analyzed by FACS with intracellular IFN-γ staining. **(D)** Gating strategy is shown in right panel, WBC gated population were further gated with CD4^+^ and CD3^+^ or CD8^+^ and CD3^+^. Staining of IFN-γ is shown in the histogram. Numbers indicate percentage of IFN-γ staining positive cells in CD4^+^ T cells or CD8^+^ T cells. Representative data from two independent experiments are shown. Numbers indicate percentage of IFN-γ staining positive cells in CD4^+^ T cells or CD8^+^ T cells. **(E)** Data are expressed as average ± SD. *N* = 6. *P < 0.05, **P < 0.01.

Depletion of CD8^+^ T cells with antibodies to CD8α or CD8β (7) increased parasitemia in PyNL infection, with ~50% mortality (Figures 1B,C). CD4^+^ T cells are essential for control of many infectious diseases and cancer. In malaria infection, subpopulations of CD4^+^ T cells have multiple roles, for example, Th1 cells help activation of macrophages and CD8^+^ T cells; Th2 cells help antibody production; regulatory T cells (Treg) and IL-27 producing CD4^+^ T cells down regulate immune cells (2, 4, 30–32). Depletion of CD4^+^ T cells was similar, with ~50% mortality (7). This suggests that both CD8^+^ T cells and CD4^+^ T cells contribute to protection against PyNL infection. IFN-γ plays an important role in protection against malaria infection (33). The spleen is an important organ in blood stage malaria infection (34, 35). Therefore, the spleens of infected mice were removed and cells were lysed, and the amount of IFN-γ produced per spleen was compared by ELISA in the presence or absence of CD4^+^ or CD8^+^ T cells (Figure 2A). First, IFN-γ was only slightly produced in non-infected mice with or without CD8^+^ T cells. Seven days after PyNL infection, IFN-γ was heavily produced, with a modest decrease in CD8^+^ T cell-depleted mice (*P* < 0.05). However, spleens from infected mice depleted of CD4^+^ T cells produced only a small amount of IFN-γ. In contrast, at 17 days post-infection, which is near the peak of parasitemia, IFN-γ was further increased in the control group, with a significant decrease in the CD8^+^ T cell-depleted infected group relative to control infected mice (*P* < 0.01, Figure 2B). In the CD4^+^ T cell-depleted group, the level of IFN-γ was not significantly different than that of the infected control group.

These results suggest that the absence of CD8^+^ T cells reduced spleen production of IFN-γ in the late stage of infection, and that the absence of CD4^+^ T cells dramatically reduced the production of IFN-γ in the early stage of infection. Recovery of IFN-γ production at the late stage of infection in the CD4^+^ T cell-depleted group was presumed to be due to complementation by other cells.

Next, we directly compared the IFN-γ producing CD4^+^ and CD8^+^ T cells in PyNL infection around peak parasitemia. T cells from spleens of uninfected or infected mice (day 16 after PyNL inoculation), were assessed for intracellular IFN-γ by flow cytometry (Figures 2C–E). The percentages of IFN-γ positive CD3^+^ CD4^+^ or CD8^+^ cells in uninfected mice were <0.4% while in infected animals, IFN-γ positivity was around 3 and 9% for CD4^+^ and CD8^+^ T cells, respectively (Figures 2D,E). The ratio of IFN-γ positive CD8^+^ T cells in infected mice was about three-fold more than that of CD4^+^ T cells. These results suggested that CD4^+^ T cells are an important source of IFN-γ early after infection while CD8^+^ T cells contribute to IFN-γ production during peak parasitemia in PyNL infection.

### Fluctuations in Spleen Cytokines During PyNL Infection in T Cell-Depleted Mice

**Figure 3:**
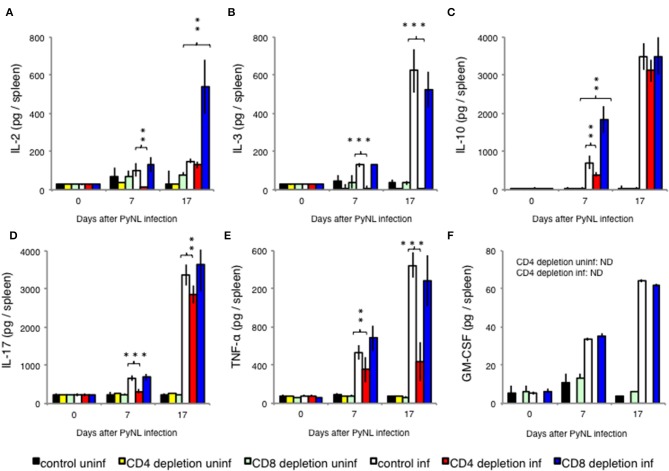
Cytokine fluctuations in spleens from CD4^+^ or CD8^+^ T cell-depleted mice *ex vivo*. CD4^+^ or CD8^+^ T cells were depleted with antibody administration 1 day before and 15 days after PyNL infection. Spleens were collected on days 0, 7, and 17 after infection. Cells were lysed and centrifuged to remove cell debris, and supernatants were subjected to ELISA (**A**: IL-2, **B**: IL-3, **C**: IL-10, **D**: IL-17, **E**: TNF-α, **F**: GM-CSF). **(E)** One representative result from four independent experiments is shown, and data are expressed as average ± SD. *N* = 3–6. **P < 0.01, ***P < 0.001.

In addition to IFN-γ, other cytokines such as IL-2, IL-3, IL-10, IL-17, and TNF-α are produced by CD8^+^ T cells stimulated by antigen presentation (36–38). These cytokines are also produced by CD4^+^ T cells (39). Therefore, we measured the amount of cytokine per mouse spleen with and without CD8^+^ or CD4^+^ T cells as in the above experiment (Figure 3). Very low amounts of these cytokines were detected in uninfected mice with and in mice without CD8^+^ T cells.

With the exception of IL-2, cytokine production increased with infection regardless of the presence or absence of CD8^+^ T cells. When CD8^+^ T cells were depleted, the amount of IL-10 produced 7 days after infection (Figure 3C) and the amount of IL-2 produced 17 days after infection (Figure 3A) significantly increased relative to the infected control group. Meanwhile, depletion of CD4^+^ T cells did not increase cytokine production relative to the infected control group. However, CD4^+^ T cell depletion decreased production of IL-2 (Figure 3A) and IL-10 (Figure 3C) in early stage infection, decreased production of IL-17 (Figure 3D) and TNF-α (Figure 3E), and abolished IL-3 production in both early and late stage infection (Figure 3B). GM-CSF is not produced by T cells, and was used as a negative control (Figure 3F).

### IL-10 Producing CD3^−^ Cells Were Increased in CD8^+^ T Cell-Depleted PyNL Infected Mice

**Figure 4:**
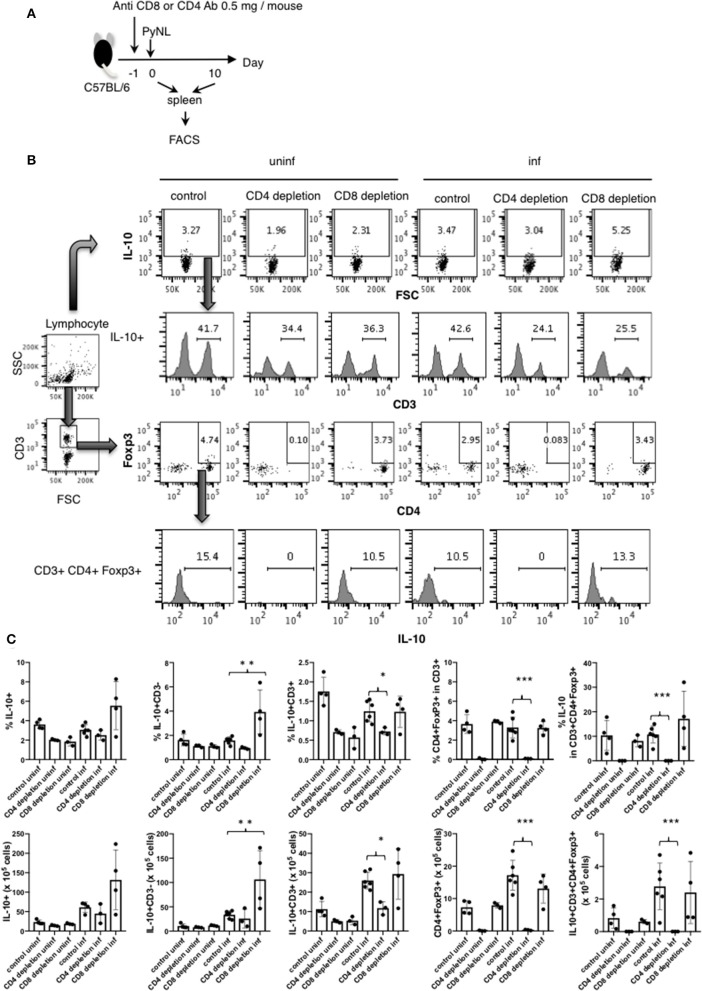
IL-10 producing CD3^−^ cells were increased in CD8^+^ T cell-depleted PyNL-infected mice. **(A)** C57BL/6 mice were injected with anti-CD8 Ab, anti-CD4 or control Ab (0.5 mg/mouse) 1 day before PyNL infection. Spleens were collected on days 0 or 10 after infection and single cells were stained with fluorescence-conjugated anti-CD3, -CD4, -CD8, -Foxp3, and -IL-10 then analyzed by FACS. **(B)** Gating strategy is shown in right panel. Lymphocytes were gated and IL-10 staining is shown in top panel. Numbers indicate IL-10 positive cells. IL-10^+^ cells were further expanded by CD3 (2nd line from top), numbers indicate percentage of CD3^+^ cells in the IL-10^+^ population. The lymphocyte gated population was gated with CD3 and then expanded for Foxp3 and CD4, numbers indicate Foxp3^+^ CD4^+^ cells in the CD3^+^ population (3rd line from top). IL-10 histogram of gated cells and number indicate percentage of IL-10^+^ cells in the CD3^+^ CD4^+^ Foxp3^+^ population (bottom panel). **(C)** Percentage or number of indicated cells are shown as average ± SD. *N* = 3–6. *P < 0.05, **P < 0.01, ***P < 0.001.

As shown in Figure 3C, splenic IL-10 production in the CD8^+^ T cell-depleted infected group was increased on day 7 after PyNL infection but not on day 17, suggesting that a more suppressive environment was formed in this cohort. However, when we looked at parasitemia on day 7 after the PyNL infection, there was no significant difference between control and CD8^+^ T cell-depleted groups until day 11, when a difference became evident (Figure 1B). Thus, we examined the flow cytometry on day 10, as this was the time point at which we speculated that a difference in IL-10-producing cells between control and CD8^+^ T cell-depleted groups could be seen. A difference in parasitemia between the control and CD8^+^ T cell-depleted group at day 10 after PyNL infection was also observed (data not shown). We then tried to identify the source of IL-10 and detect Treg cells. Spleen cells from CD4^+^ or CD8^+^ T cell-depleted mice with or without PyNL infection (day 10 post infection; p.i.) were analyzed by flow cytometry (Figure 4A). The percentage and number of IL-10^+^ cells in the CD8^+^ T cell-depleted infected group appeared to be higher than the control infected group but this difference was not statistically significant. When we looked at IL-10^+^ CD3^−^ cells in the CD8^+^ T cell-depleted mice, the percentage and number were increased two-fold more than the control-infected group, while no differences in IL-10^+^ CD3^+^ cells, Foxp3^+^ CD4^+^ T cells, or IL-10^+^ Foxp3^+^ CD4^+^ T cells were found (Figures 4B,C). From this result, the CD8^+^ T cell-depleted infected group did indeed show more IL-10^+^ CD3^−^ cells in the spleen on day 10 after PyNL infection, which might contribute to formation of the immunosuppressive environment and result in subsequent hyperparasitemia and death.

### Lactate Is One of the Causes of Death in CD8^+^ T Cell-Depleted PyNL-Infected Mice

**Figure 5:**
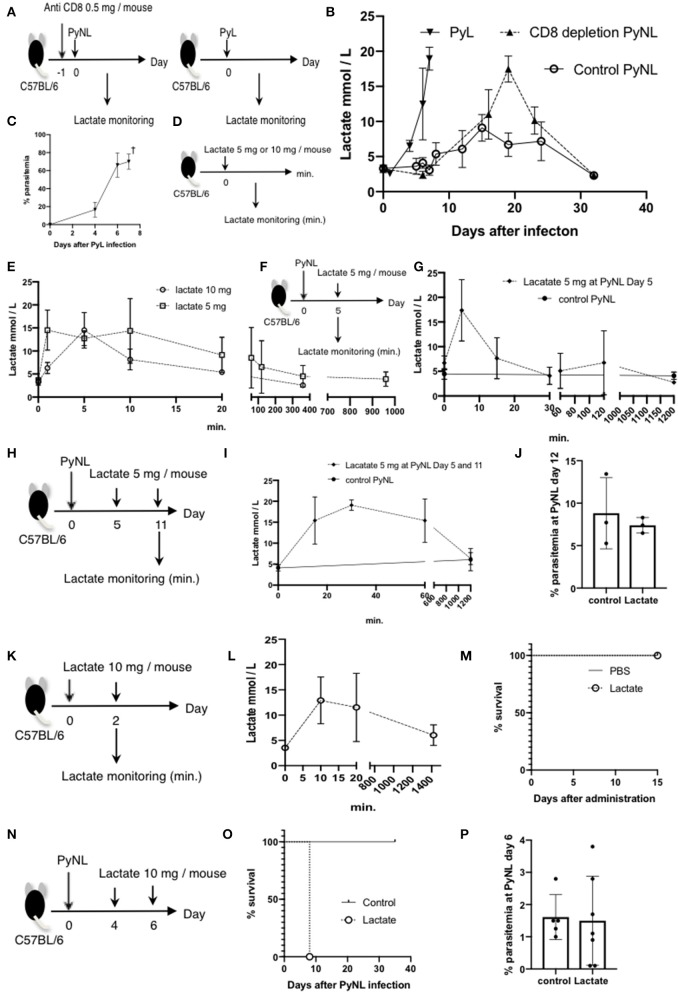
Lactate is one of the causes of death in CD8^+^ T cell-depleted PyNL-infected mice. **(A)** C57BL/6 mice were injected with anti-CD8 Ab or control Ab (0.5 mg/mouse) 1 day before PyNL infection and blood lactate was measured (right panel). C57BL/6 mice were infected with PyL and blood lactate was measured (left panel). **(B)** Fluctuations in blood lactate in PyL-infected (*N* = 7), CD8^+^ T cell-depleted PyNL-infected (*N* = 3), and control PyNL-infected mice (*N* = 17) are shown. Data are pooled from three independent experiments. **(C)** Changes in parasitemia of PyL infected mice are shown. All mice died at day 8 p.i. **(D,E)** C57BL/6 mice were administered 5 or 10 mg of lactate and blood lactate was monitored. *N* = 3. **(F,G)** C57BL/6 mice were infected with PyNL, 5 mg of lactate was administrated on day 5 p.i. and blood lactate was monitored. Control PyNL: *N* = 6, lactate 5 mg PyNL: *N* = 3. **(H,I)** C57BL/6 mice were infected with PyNL and 5 mg of lactate was administered on day 5 and 11 p.i. and blood lactate was monitored. Control PyNL: *N* = 6, lactate 5 mg PyNL: *N* = 3. **(J)** Percentages of parasitemia of mice from experiment H on day 12 p.i. are shown. **(K,L)** C57BL/6 mice were administered 10 mg of lactate on day 0 and 2 and blood lactate was monitored. *N* = 3. **(M)** Survival rate of mice from experiment K is shown. **(N)** C57BL/6 mice were infected with PyNL and 10 mg of lactate was administered on day 4 and 6. **(O)** Survival rate and **(P)** percentage of parasitemia of mice from experiment N are shown. Control: *N* = 5; 10 mg lactate: *N* = 7.

Blood lactate concentration is the one of the root causes of disease severity in *P. falciparum* (40). We investigated whether lactate is related to pathogenesis of Py infection or not. First, we measured the blood lactate concentration in PyL and PyNL infected mice (Figures 5A,B). Blood lactate in PyL infected mice was elevated from 2 to 4 mmol/L before infection to 19 mmol/L at day 7 p.i. (Figure 5B) while parasitemia increased to 80% (Figure 5C). All mice died at day 8 after PyL infection. Blood lactate of PyNL infected CD8^+^ T cell-depleted mice was also elevated and reached 17.5 mmol/L around the time of peak parasitemia (60%) at day 19, while that of the control PyNL-infected group was 9.1 mmol/L at day 15. Upon clearance of the parasites, blood lactate in both of these groups decreased to the same level as that in uninfected. This result indicated that PyL-infected mice demonstrate the same tendency of peak hyperlactatemia and peak hyperparasitemia as CD8^+^ T cell-depleted PyNL infection. Next, we tried to reproduce the hyperlactatemia by injecting lactate. We administered 5 or 10 mg of lactate i.p. to uninfected mice and monitored the blood lactate concentration (Figure 5D), which peaked within 10 min in both 5 and 10 mg lactate injection groups. Interestingly, there was no difference in peak concentration, which was 15 mmol/L in both 5 and 10 mg groups, and this was metabolized to normal concentrations within 400 and 1,000 min, respectively (Figure 5E). We examined lactate clearance by injecting 5 mg of lactate into PyNL-infected mice at day 5 p.i. (Figure 5F). Blood lactate concentration was increased to 17 nmol/L from 4.5 nmol in 5 min after administration and decreased to base line within 30 min (Figure 5G). This indicates that PyNL infected mice can metabolize 5 mg of injected lactate. We assumed that a single lactate injection was not enough to reproduce the hyperlactatemia caused by PyL or CD8^+^ T cell-depleted PyNL infection thus we injected 5 mg of lactate again at day 11 p.i. with PyNL (Figure 5H). Peak blood lactate concentration was delayed to 30 min post injection and remained elevated at 60 min (15 mmol/L) before being metabolized within 1,200 min.

We tried to elucidate the relationship between lactatemia and parasitemia, thus we investigated the parasitemia, however, there were no differences in parasitemia or in survival rate between mice with or without lactate injection. We therefore assumed that 5 mg was insufficient to induce hyperlacatatemia and thus tested 10 mg lactate injected twice into uninfected mice (Figure 5K). The result was not the hyperlactatemia that we expected, with a lactate peak concentration of 13 mmol at 10 min which was metabolized in 1,400 min (Figure 5L). There was no effect on survival of uninfected mice following two 10 mg lactate injections (Figure 5M). Finally, we tested the effect of two 10 mg lactate injections into PyNL-infected mice at day 4 and 6 p.i. (Figure 5N). Surprisingly, all mice died at day 8 (Figure 5O), while there was no difference in parasitemia between mice with or without lactate injection. This result suggested that malaria infection plus high lactate administration caused death despite low parasitemia (Figure 5P). These data suggest that hyperlactatemia might be one of the causes of death of malaria infection which we observed in PyL and CD8^+^ T cell-depleted PyNL infected mice.

### Immunoproteasome Is Not Required for Induction of CD8^+^ T Cell in the Live Vaccine Model

**Figure 6:**
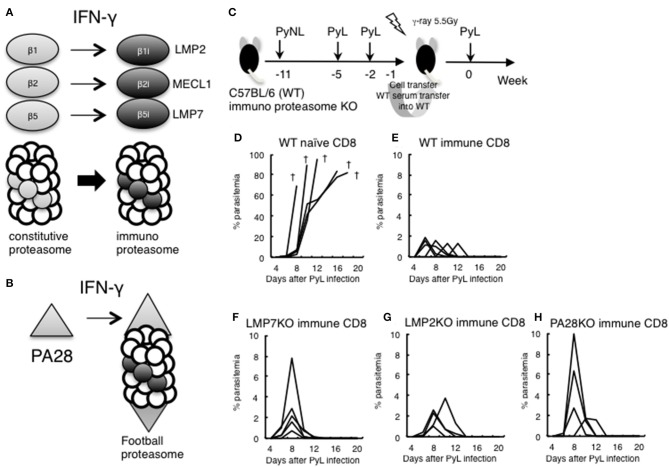
Evaluation of immunoproteasome in CD8^+^ T cell induction in live vaccine model. **(A)** IFN-γ converts subunits β1, 2, and 5 from the constitutive proteasome into β1i (LMP2), β2i (MECL1), and β5i (LMP7) to construct the immunoproteasome. **(B)** Proteasomal cap structure PA700 is converted to PA28 by IFN-γ, and PA28 binds to the immunoproteasome to construct a football-like proteasome. **(C)** Live vaccine model against PyL infection. Wild type (WT) and various KO mice were inoculated with PyNL and PyL infected RBCs at the indicated time points, and CD8^+^ T cells (1 × 10^7^ cells) from immunized WT donor mice or naïve WT mice were transferred into irradiated (5.5 Gy) WT recipient mice, followed by challenge with PyL infection in recipient mice. Parasitemia of recipient mice receiving donor immune CD8^+^ T cells from **(D)**: WT naïve, **(E)**: WT infected, **(F)**: *LMP7*^−/−^, **(G)**: *LMP2*^−/−^, **(H)**: *PA28*^−/−^. Each line represents a value from an individual mouse (*N* = 4–6). Dagger symbols indicate death. Similar results were obtained from two independent experiments.

The above results suggested that IFN-γ was not sufficiently produced in the late stage of infection in CD8^+^ T cell-depleted mice. IFN-γ converts the subunits of the constitutive proteasome β1, 2, and 5 into β1i (LMP2), β2i (MECL1), and β5i (LMP7) to construct the immunoproteasome (Figure 6A). Furthermore, the proteasomal cap structure PA700 is converted to PA28, and PA28 binds to the immunoproteasome to construct a football-like proteasome (Figure 6B) (22). These conversions are thought to enhance antigen presentation to MHC class I molecules.

We previously developed a DNA vaccine against tumors and parasites (*Toxoplasma, Trypanosoma*) and have found that the efficacy of the DNA vaccine was abrogated when we used *LMP7*^−/−^, *LMP2*^−/−^, and *PA28*^−/−^. The results suggest that the immunoproteasome and/or PA28 is involved in the induction of antigen-specific CD8^+^ T cells by DNA vaccine against tumors and parasites (41–46).

In order to determine whether CD8^+^ T cell induction depended on the immunoproteasome and PA28 in the malaria live vaccine model, WT and various KO donor mice were inoculated with PyNL and PyL infected RBCs, and CD8^+^ T cells from immunized donor mice were transferred into irradiated WT recipient mice. We then examined protection against PyL infection challenge in recipient mice (Figure 6C). The WT naive CD8^+^ T cell recipient group was unable to develop immunity to PyL, and all mice rapidly developed parasitemia and died (Figure 6D). Contrastingly, inoculation with activated WT immune CD8^+^ T cells conferred protective immunity against PyL in recipient mice, with 2% or less of maximum parasitemia (Figure 6E) (7, 16). Immune CD8^+^ T cells derived from *LMP7*^−/−^ (Figure 6F), *LMP2*^−/−^ (Figure 6G), and *PA28*^−/−^ (Figure 6H) mice also induced protective immunity in recipient mice, although the maximum parasitemia was at least two-fold higher than WT. These findings suggest that single ablation of immunoproteasome factors did not impair induction CD8^+^ T cells.

### Fas Contributes to Protection Against PyNL Infection

**Figure 7:**
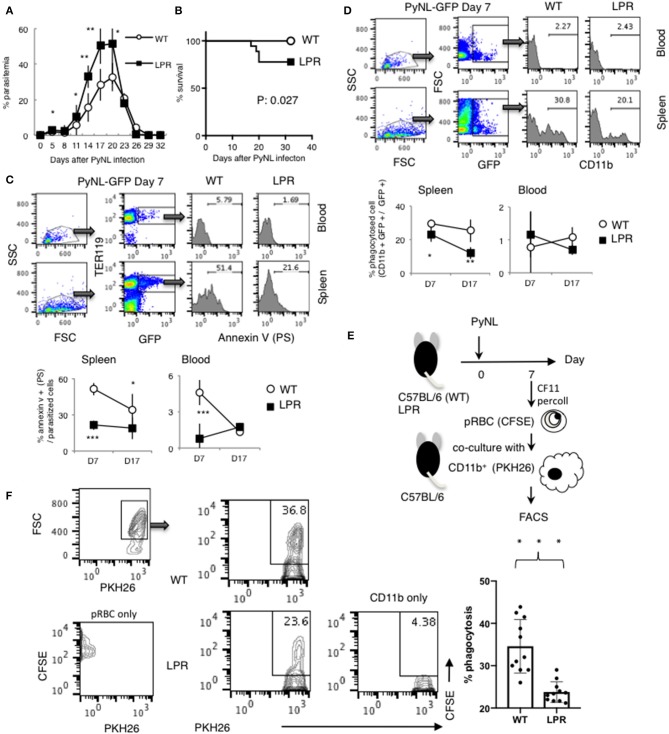
Fas contributes to protection against PyNL infection. **(A)** Parasitemia and **(B)** survival rate are shown. Parasitemia values are expressed as average ± SD. Data are representative of two pooled independent experiments (WT: *N* = 19, LPR: *N* = 14). **(C)** PyNL-GFP was inoculated on day 0, and spleen and peripheral blood were collected at day 7 and 17 after infection for analysis of PS externalization using annexin V and Ter119 (erythroid cell marker) and GFP flow cytometric analysis. One example of analysis in PyNL-GFP at day 7 is shown (Top panel). WBC gated population with dot plot of FSC and SSC were further gated for TER119^+^ GFP^+^ infected erythroid cells and expand with Annexin V (PS). Numbers indicate percentage of PS^+^ cells in infected erythroid cells in blood or spleen. Data are expressed as average ± SD from two pooled independent experiments (bottom panel, WT: N = 11, LPR: *N* = 9). **(D)** Percent phagocytosed cells (proportion of CD11b^+^ GFP^+^ in GFP^+^ cells) was determined by flow cytometry. One example of analysis in PyNL-GFP day 7 is shown (Top panel). WBC gated population with dot plot of FSC and SSC were further gated for GFP^+^ cells which include infected cells and phagocytosed cells and expand with CD11b. Numbers indicate percentage of CD11b^+^ cells in GFP^+^ cells in blood or spleen. Data are expressed as average ± SD from two pooled independent experiments (bottom panel, WT: *N* = 11, LPR: *N* = 9). **(E)** Parasitized RBCs (pRBCs) were isolated from peripheral blood 7 days after infection and labeled with CFSE for the phagocytosis assay. CD11b^+^ macrophages isolated from uninfected mice were labeled with PKH26, co-cultured with pRBCs and analyzed by flow cytometry to determine the phagocytosis rate. **(F)** One representative result (left panel) and individual data are expressed as % phagocytosis = PKH^+^ CFSE^+^ / PKH^+^ (WT: *N* = 11, LPR: *N* = 11) from three pooled independent experiments. *P < 0.05, **P < 0.01, ***P < 0.001.

The Fas-FasL interaction induces apoptosis of the Fas expressing cell, which is characterized by PS externalization of the cell surface and morphological changes, among others (47–50). We previously reported that Py infection induced enhancement of FasL on CD8^+^ T cells but not on CD4^+^ T cells. FasL mutant GLD mice were more susceptible to PyNL infection than WT mice, exhibiting increased parasitemia and decreased survival rate. Consistent with this, in our PyNL and PyL live vaccination model, immune CD4^+^ T cells did not require FasL for protection against PyL challenge, but immune CD8^+^ T cells were partially dependent on FasL (7). Thus, the Fas-FasL pathway seems to be important for CD8^+^ but not CD4^+^ T cells against blood stage malaria infection. In this study, we focused on Fas, to confirm the importance of the Fas-FasL pathway in host defense against PyNL. Ohno et al. previously reported that the C57BL/6 background LPR mouse has reduced resistance to PyNL, but the mechanism has not been clarified (51). In the present study, we re-investigated the course of infection in LPR mice infected with PyNL. In infected WT mice, parasites were eliminated after reaching peak parasitemia ~20 days after infection (Figure 7A). Contrastingly, parasitemia was significantly exacerbated in infected LPR mice, with increased parasitemia at the peak time of 17–20 days and a 77% survival rate after infection (Figures 7A,B). We therefore reproduced the data reported by Ohno et al. and tried to reveal the mechanisms responsible.

Because externalization of PS caused by the Fas-FasL interaction triggers phagocytosis by macrophages, this process is an important protective mechanism induced by CD8^+^ T cells in blood stage malaria infection (7). We assumed that the externalization of PS on infected erythroid cells is reduced in LPR compared with WT mice during PyNL infection and reflects the differences in parasitemia. Therefore, LPR mice were infected with PyNL-GFP, and externalization of PS on infected erythroid cells (Ter119^+^) was analyzed by flow cytometry. Many parasitized erythroblasts are present in the spleen and bone marrow, whereas almost none are present in peripheral blood. The rate of PS on the surface of parasitized erythroid cells (Ter119^+^ GFP^+^) was evaluated by staining with AnnexinV, which binds to PS (Figure 7C). Seven days after infection, PS-displaying parasitized erythroid cells were 51% in WT spleen, compared with 21% in LPR spleen (*P* < 0.001). Further, 17 days after infection, 19% of spleen cells displayed PS in LPR mice, compared to 34% in WT mice (*P* < 0.05). In peripheral blood seven days after infection, PS-displaying cells were 4.6% in WT and 0.8% in LPR mice (*P* < 0.05), whereas at 17 days post-infection, there was no significant difference between WT (1.3%) and LPR (1.7%) mice. Taken together, these findings suggested that the proportion of cells displaying PS was higher in WT parasitized erythroid cells than in LPR parasitized erythroid cells, which might be due to the interaction between Fas and FasL.

Next, the phagocytosed rate (proportion of CD11b^+^ GFP^+^ in GFP^+^ cells) was examined (Figure 7D). In peripheral blood, almost no parasitized erythroid cells were phagocytosed, and there was no difference between WT and LPR mice. In the spleen, seven days after infection, the rate of phagocytosis in was 29 and 21% in WT and LPR mice, respectively (*P* < 0.05). Seventeen days post-infection, the rate of phagocytosis was 25 and 12% in WT and LPR mice, respectively (*P* < 0.001). Parasitized cells were phagocytosed more efficiently in WT than LPR mice. In *in vivo* experiments, phagocytosis could be altered not only by the condition of parasitized erythroid cells, but also by the activation of macrophages. Therefore, we used an *in vitro* approach to evaluate whether PS externalization and phagocytic rate were correlated. There are technical limitations in utilizing spleen-derived parasitized erythroblasts, and therefore parasitized RBCs were isolated from peripheral blood 7 days after infection and labeled with CFSE for the phagocytosis assay (Figure 7E). At this time point, pRBCs from WT mice were more likely to display PS on the surface. WT CD11b^+^ macrophages were then isolated from uninfected mice and labeled with PKH26, co-cultured with pRBCs and analyzed by flow cytometry to determine the phagocytosis rate. The phagocytosis rate was ~35% in WT pRBCs and ~24% in LPR pRBCs, suggesting that macrophages more efficiently phagocytosed RBCs from WT animals than from LPR animals (Figure 7F, *P* < 0.05). These results suggested that PS externalization leads to the phagocytosis of parasitized erythroid cells by macrophages and is related to parasitemia, which was dependent on the Fas-FasL pathway.

### MFG-E8 Does Not Contribute to Host Defense Against PyNL Infection

**Figure 8:**
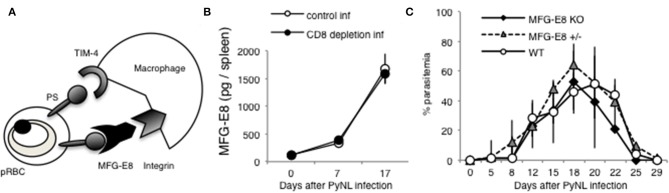
MFG-E8 is not critical for host defense against PyNL infection. **(A)** PS is externalized on some pRBCs, and recognized by TIM-4 and MFG-E8. MFG-E8 binds with macrophage integrin. **(B)** Spleens were collected from day 0, 7, and 17 after infection. Cells were lysed and centrifuged to remove cell debris, and supernatant was subjected to ELISA (MFG-E8). Data were obtained from two pooled independent experiments, and are expressed as average ± SD. *N* = 6. **(C)** MFG-E8-KO (-/- and +/- mice) mice were infected with PyNL. Parasitemia is shown (*MFG-E8*^−/−^: *N* = 7, *MFG-E8*^+/−^: N = 6, WT: *N* = 7). Data were obtained from two pooled independent experiments.

Tim-4 and MFG-E8 are known to bind PS [(25, 52), Figure 8A]. A previous study identified that antibodies against Tim-4 partially inhibited phagocytosis *in vitro* (7). For the present study, we focused on MFG-E8. Splenocyte MFG-E8 was increased with PyNL infection, and there was no difference with or without CD8^+^ T cells (Figure 8B). Next, *MFG-E8*^−^^/−^ mice were infected with PyNL. Both heterozygote and KO mice had the same infection course as WT, suggesting that MFG-E8 was not involved in protection against PyNL infection (Figure 8C). We are interested in determining if the binding of MFG-E8 to PS can promote phagocytosis of pRBCs.

## Discussion

In this study, we examined cytokines from CD8^+^ T cells and CD4^+^ T cells in blood stage PyNL infection, the death receptor Fas, phagocytosis by macrophages, and the involvement of MFG-E8, which is a PS-binding molecule. In the early stage of PyNL infection, CD8^+^ T cell-depleted C57BL/6 mice had increased splenic production of IL-10 relative to control infected mice. In the late stage of infection, CD8^+^ T cell-depleted mice had decreased production of IFN-γ and increased production of IL-2. In the CD4^+^ T cell-depleted group, production of IL-2 and IL-10 were decreased in the early stage of infection, and production of IL-17 and TNF-α was decreased in both the early and late stages. Further, production of IL-3 was lost in CD4^+^ T cell-depleted animals. In blood-stage PyNL infection, CD4^+^ T cells seem to be more important for cytokine production, and CD8^+^ T cells appear to be responsible for cytotoxic activity and IFN-γ production as killer T cells. The LPR mouse had decreased resistance to PyNL infection, with a 20% mortality rate. LPR-derived parasitized erythroid cells had decreased PS externalization, and were not phagocytosed as efficiently. MFG-E8 production increased with infection, but the course of infection in *MFG-E8*^−/−^ mice was similar to that of WT mice, suggesting MFG-E8 did not contribute in a major manner to protective immunity against PyNL.

Prior reports have identified that PyNL and PyL infection alter production of cytokines in spleen and liver (53, 54). IFN-γ has various functions, including macrophage activation and induction of MHC class I and Fas expression (55). We have also confirmed that IFN-γ increases expression of MHC class I on erythroblasts when added to erythroblasts *in vitro* (21). The concentration of IFN-γ is increased in blood with Vivax malaria infection (56), and IFN-γ is an important cytokine in both blood stage malaria protection and pathology (3, 33, 57, 58).

A prior study has demonstrated that CD8^+^ T cells are activated 7 days after PyNL infection, expressing CD25, Fas, LAMP1, and other immune regulators (7), but the ability of CD8^+^ T cells to produce IFN-γ is diminished. Conversely, CD4^+^ T cells do not express Fas after infection (7), and have increased IFN-γ production. In fact, because spleen IFN-γ was reduced to the same level as non-infected mice on day 7 after infection in CD4^+^ T cell-depleted mice, most IFN-γ may be produced by CD4^+^ T cells, at least in the early stage of infection. Contrastingly, at the late stage, there was no difference between the CD4^+^ T-cell depleted group and the control infected group, potentially due to compensation by other cell types. However, in late stage infection, IFN-γ production was decreased in CD8^+^ T cell-depleted animals. Indeed, the IFN-γ positive CD8^+^ T cells in spleen of PyNL infected mouse was about three-fold more than that of CD4^+^ T cells in day 16 after the infection (Figure 2). This suggests that IFN-γ production is mediated by CD8^+^ T cells in late-stage infection. Other immune cells present in CD8^+^ T cell-depleted animals did not compensate to produce a sufficient amount of IFN-γ for protection against infection. Meanwhile, CD8^+^ T cells do not seem to require IFN-γ in protection against liver stage malaria infection (59). Therefore, the importance of IFN-γ may differ by infection stage. In any case, both CD4^+^ T cells and CD8^+^ T cells are thought to contribute to host defense by producing IFN-γ in blood stage malaria infection.

Another factor downstream of IFN-γ is the induction of the immunoproteasome. We previously demonstrated that the immunoproteasome is involved in the induction of antigen-specific CD8^+^ T cells. Accordingly, *the LMP7*^−/−^ mouse is more susceptible to tumors (44), *Toxoplasma* (60) and *Trypanosoma* (61) infection. Therefore, we infected *LMP7*^−/−^ mice with PyNL or PyL, and unexpectedly found that *LMP7*^−/−^ mice were more resistant than WT against infection (62). The production of IFN-γ in CD8^+^ T cells from *LMP7*^−/−^ mice was reduced during infection. Various investigations revealed that resistance against infection was caused by changes in the structure of parasitized RBCs in *LMP7*^−/−^ mice, making parasitized RBCs more susceptible to phagocytosis in these animals.

In the present study, we conducted infection experiments in *LMP2*^−/−^ and *PA28*^−/−^ mice in addition to *LMP7*^−/−^ mice (Figure 4). Unexpectedly, these KO mice were more resistant to primary infection by PyL than WT mice (data not shown). We examined whether immune CD8^+^ T cells induced in a live vaccine model of PyL are dependent on immunoproteasome-related factors (Figure 4). In cell transfer experiments, recipient mice that received CD8^+^ T cells derived from immunized immunoproteasome-related factor KO mice were slightly less resistant to PyL infection than recipient mice that received CD8^+^ T cells derived from WT mice. However, all mice were able to develop protective immunity against PyL infection, thus suggesting that at least the single molecules of LMP7, LMP2, and PA28 are not essential for induction of CD8^+^ T cells.

IL-10 is involved in preventing the onset of experimental cerebral malaria (ECM) (63). At the early stage of PyNL infection in CD4^+^ T cell-depleted mice, IL-10 production was reduced compared to the control infected group, but at later stages of infection, other cell types compensated for this effect and IL-10 production in CD4^+^ T cell-depleted mice was similar to that of control infected mice. Contrastingly, in the CD8^+^ T cell-depleted infected group, IL-10 production was increased relative to control infected animals, similar to IL-2. Early stage IL-10 overproduction may be linked to decreased late stage IFN-γ production.

FasL-Fas is important for CD8^+^ T cell-mediated protection from PyNL infection. In the present study, we examined this relationship using Fas mutant LPR mice (Figure 5). LPR mice were less resistant to PyNL infection than WT (51), further confirming this finding. This is likely because the cell death signal from CD8^+^ T cells to parasitized cells is not transmitted properly and PS expression was poor, impairing phagocytosis by macrophages. This finding suggests that target cell Fas signaling also contributes to host defense against PyNL infection. However, in the phagocytosis study using parasitized RBCs from LPR mice, phagocytosis is less efficient but still occurs, suggesting that other mechanisms also contribute to phagocytosis. Stimulation on Fas suppresses uptake of the energy source glucose, which also leads to cell death (64). Therefore, even if malaria parasites are not phagocytosed, glucose supply to the protozoa in parasite cells may be insufficient, resulting in parasitic cell death. MHC class I and Fas are expressed in parasitized erythroblasts, but Fas expression is not observed in parasitized RBCs (7). However, parasitized erythrocytes externalize PS, which is dependent on CD8^+^ T cells. We assumed that parasitized erythroblasts were attacked by CD8^+^ T cells simultaneously or later enucleated, maturing into RBCs with externalized PS in the peripheral blood.

In a series of studies including the present report, we hypothesized that IFN-γ from CD8^+^ T cells up-regulates MHC class I and Fas expression on parasitized erythroblasts (7, 21). Subsequently, cytotoxic activity of CD8^+^ T cells leads to externalization of PS in parasitized erythroid cells, promoting phagocytosis by macrophages. Phagocytes appear to be the most important cells in protection against blood stage malarial infection, and all mice die with PyNL infection when macrophages are depleted (7, 16).

A recent report found that in humans, MHC class I molecules on RBCs infected with *P. vivax* trigger CD8^+^ T cell recognition and subsequent cell death mediated by cytotoxic molecules such as granulysin (65). This finding supports a protective role for CD8^+^ T cells in blood stage malaria. In our system, it was not possible to induce PS externalization by CD8^+^ T cells or FasL in parasitized RBCs (7). Although we have not examined parasite survival, mouse malaria may not be the same as human, as malaria infection does not induce granulysin expression in mice. This suggests that a vaccine that not only induces antibody production but also activates CD8^+^ T cells may be able to confer protection against *P. vivax*. We have already developed a live vaccine model in mice, and demonstrated that transfer of immunized CD8^+^ T cells to recipient mice can protect against lethal malaria strains (16).

**Figure 9:**
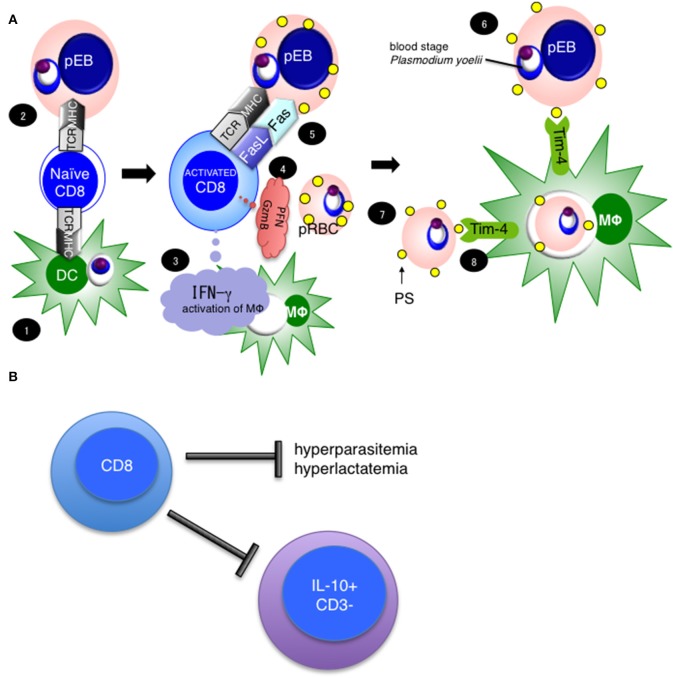
Hypothesis and importance of CD8^+^ T cell mediated protection against blood stage malaria. **(A)** Our current hypothesis is based on this study and three previously published papers, cited in the main text. (1) CD8^+^ T cells are activated with antigen cross-presentation from DC or (2) direct antigen presentation from parasitized erythroblasts, resulting in CD8^+^ T cell IFN-γ Perforin (PFN) and Granzyme B (GzmB) production. (3) IFN-γ activates macrophages. (4) Meanwhile, CD8^+^ T cells recognize MHC class I on parasitized erythroblasts and stimulate through FasL to (5) Fas on erythroblasts, then (6) induce the externalization of phosphatidylserine (PS) to the surfaces of parasitized erythroblasts. (7) The externalization of PS is a feature of apoptotic cells, and (8) leads to phagocytosis by macrophages. Tim-4 is involved in this process as a PS receptor. **(B)** CD8^+^ T cells regulate hyperparasitemia, hyperlactatemia and the number of IL-10^+^ CD3^−^ cells to protect against PyNL infection.

We have reported that CD8^+^ T cells are important for protection against blood stage malaria infection (7, 16). Through this research, we found that rodent malaria parasites infect erythroblasts, which are the precursor cells for red blood cells (RBCs) (21). Currently, we hypothesize that CD8^+^ T cells protect against blood stage malaria infection in the steps summarized in Figure 9A. We suggest that (1) CD8^+^ T cells are activated by cross-presentation from DCs or (2) direct presentation from parasitized erythroblasts, and once activated produce IFN-γ Perforin (PFN) and Granzyme B (GzmB). (3) Subsequently, IFN-γ activates macrophages, and up-regulates expression of MHC class I molecules (55). (4) Meanwhile, CD8^+^ T cells recognize MHC class I on parasitized erythroblasts, and stimulate FasL to (5) Fas on the erythroblasts, then (6) induce externalization of phosphatidylserine (PS) on the surface of parasitized erythroblasts. (7) The externalization of PS is a feature of apoptotic cells, and (8) leads to phagocytosis by macrophages. Tim-4 is involved in this process as a PS receptor. Additionally, we hypothesize that CD8^+^ T cells regulate not only hyperparasitemia but also hyperlactatemia and regulate the IL-10^−^ CD3^−^ cells (Figure 9B). We believe that hyperlactatemia is one of the causes of death in malaria and indeed, control of hyperlactatemia is important for saving *Plasmodium falciparum* patients (40).

Furthermore, we are currently developing ubiquitin fusion DNA vaccines that efficiently induce antigen specific CD8^+^ T cells, and have demonstrated protective effects against tumors (29, 41, 42, 44, 46), *Trypanosoma* (45), and *Toxoplasma* (43). If the mechanisms for CD8^+^ T cell-mediated protection from malaria can be analyzed in detail, and CD8^+^ T cells can be manipulated clinically, it may be possible to selectively target parasitized cells, preventing pathogenesis of malaria.

## Data Availability

All datasets generated for this study are included in the manuscript/supplementary files.

## Ethics Statement

The animal study was reviewed and approved by The Committee for Ethics on Animal Experiments in the Faculty of Medicine, adhered to the Guidelines for Animal Experiments in the Faculty of Medicine, Gunma University and Kyushu University.

## Author Contributions

TI directed the project, planned and performed experiments, analyzed results, created figures, and wrote the manuscript. KS analyzed the results. HN-T, SOn, WO, HS, CS, AO, SOb, and TT performed the experiments. HI generated the recombinant parasite. KT and SM generated the PA28KO mice. LV generated the LMP2KO mice and revised the manuscript. HH supervised the project and critical revision of the manuscript for important intellectual content.

### Conflict of Interest Statement

The authors declare that the research was conducted in the absence of any commercial or financial relationships that could be construed as a potential conflict of interest.

## Footnotes

^1^WHO world malaria report (2018). http://www.who.int/malaria/publications/world-malaria-report-2018/en/
